# Lithontrity

**Published:** 1830

**Authors:** A. G. Smith


### II. Original.

*Lithontrity.*—In a Letter from the Senior Editor of this Journal, dated Philadelphia, Dec. 2d, 1830.

" A month has now elapsed since my arrival in this *emporium* of the Medical Sciences. I have met with many learned and able physicians, surgeons, and paturalists, but as yet have not been made acquainted with any thing new or particularly interesting in the profession. A longer sojourn may, perhaps, put me in possession of unpublished discoveries, improvements, or projects, worthy of being laid before our readers.

Meanwhile, in continuation of the efforts already made to direct their attention to the Lithontrity of Civiale, as a substitute for Lithotomy, I will inform you and them, that a new attempt to introduce it into the United States is now in progress.

Nearly a year and a half ago, Dr. Alban G. Smith, of Danville, Kentucky, determined to visit Paris, and become the pupil of Civiale, for the sole purpose of studying Lithontrity, and having made himself familiar with every thing relative to it, is now in this city on his return. Last night he showed me his instruments, and they certainly look promising, in reference to the object in view. He, himself, is fully convinced of the great utility of the operation, and feels sanguine in his expectations of introducing it into the United States. He is now on his way home, and will, at no distant time, select a situation for the scene of his efforts. Being from the West, I hope he will remain in the West.

At my request he has furnished me with the following notices relative to Lithontrity, which, from their authenticity and recent date deserve insertion in the Journal."

Case 1. A man aged 26 had been laboring under the disease five years. On his admission into the hospital he was put upon low diet ten days, and had the catheter introduced daily. M. Leroy operated on Saturday, and took up the stone at the first attempt. The patient, so far from complaining, said it did not hurt him at all ; and upon rising from the table he passed his urine with about a thimble-full of the stone. Next morning on visiting him, he said he was very comfortable, and continued so until the Saturday following, when he was operated upon again without complaining. The stone was found in two fragments, one of which was broken up ; next morning he showed a considerable portion of the stone that he had passed during the night, which he said hurt no more than when he urinated previous to the operation. On the next Saturday he was operated on again, and passed nearly all the balance of the stone during the night. He complained of some soreness in the morning. In three days it ceased to pass, and he was discharged perfectly cured.

Case 2. Another case of a man sixty years of age, was admitted at La Chartre, with a stone about 12 lines in diameter. The largest sized instrument was introduced, the stone was soft and easily broken with the hand. After each operation he passed a considerable portion of stone ; he walked to and from the hospital to be operated upon, and complained but little during the operation. It required six operations to relieve him. I saw him some weeks afterwards, when he said he had since had no symptoms of the disease.

Case 3. A child 7 years of age was operated on with one of two and a half lines in diameter. It was necessary to enlarge the meatus urinarius with a bistoury. The introduction of the instrument was very painful, and the child contracted its bladder very much, but the stone, about five lines in diameter, was seized without difficulty and broken down. The patient complained very much during the withdrawing of the instrument. During the night four large pieces were discharged with the urine. He was operated on four times more with less pain than in the first attempt, and was discharged cured.

Case 4. A child aged four years and six months was operated on with an instrument of two lines. The bladder was very small, and the stone, five or six lines in diameter, was grasped with difficulty. The rectum was inverted by the cries of the child. During the night eight grains of the calculus were discharged, and by three more operations, which were attended by nothing remarkable, the patient was entirely relieved.

*Remarks.*—It is not expected that this operation will ever come to be very generally employed by surgeons. On the contrary, the number of operators will no doubt always be smaller in proportion than that of those who now perform lithotomy. The practice necessary to enable an individual to operate successfully, is much greater than is requisite for that operation, while the facilities for acquiring the necessary skill, are much more limited. There are but two successful operators in Paris, Le Roy and Civiale. The former of whom operates in La Charite and Hotel Dieu, the latter has a ward in Hospital Neckar in which he receives his patients. There are but two also in England, one of whom is a foreigner.

With these difficulties in the way of its coming into general use with the profession at large, there can be no doubt of its having in the hands of an expert and practised operator, many advantages over lithotomy.

1. It does away all fear of a dangerous and bloody operation, and gives to the patient that confidence so favorable to a happy recovery.

2. The position of the patient during the operation is neither painful nor constrained.

3. The pain of Lithortrity, in favorable cases, and in the hands of prudent and well experienced operators, is trifling. If the pain at any one time should be very severe, the operation may be postponed, till the system is in a more favorable condition, or it may be often repeated in short sittings, to suit the condition of the patient.

4. If the operator be prudent and skilful, the operation is not liable to any dangerous accidents. The patient can generally attend to his ordinary business, and with each successive operation, the situation of the patient is improved and the pain diminished.

5. If it be urged that in many cases the operation is followed by unpleasant consequences, it is admitted that much practice is necessary to qualify the surgeon to operate, and if the attempt should fail from the want of the necessary preparation, the ill success is certainly not to be laid to the fault of the operation.

6. There are doubtless cases in which the operation cannot be resorted to. These cases, however, it is to be borne in mind, are almost eqnally unfavorable for lithotomy. During my attendance on the Parisian Hospitals, it fell to my lot to witness thirty operations with this instrument, all of which terminated favorably ; while of all the patients upon whom lithotomy was performed, on account of the impracticability of using this instrument, but one recovered. It is also to be remembered that the impracticability of using this instrument arises in many cases from the delay produced by the fear of the knife. There can be no doubt, therefore, that when the character of this mode of operating shall be so established, as to induce an early resort to it, it will be found applicable to a much larger proportion of cases. It may not be improper here to mention that the subjects of the operation met with in the United States, are generally more favorable to its success than those which are met with in the European Hospitals. It will therefore, no doubt, be a safe estimate to say that three fourths of the patients of this country are proper subjects of this instrument. It may not be improper to mention as an evidence of the estimation in which the operation is held in Paris, that it is adopted in all the hospitals in that city ; and the National Institute have recommended to the government, to appoint a distinct Lithontripteur to each hospital.

